# Congenital Myasthenia Gravis Presenting as Refractory Seizures and Respiratory Failure: A Case Report

**DOI:** 10.7759/cureus.76886

**Published:** 2025-01-03

**Authors:** Raiya AlHabsi

**Affiliations:** 1 Pediatrics, Ibra Hospital, Ibra, OMN

**Keywords:** acetylcholine receptors, congenital myasthenia syndrome, myasthenia gravis (mg), neuromuscular junction disorders, whole exome sequencing (wes)

## Abstract

Congenital myasthenia syndrome (CMS) is an inherited disorder that involves muscle weakness and fatigue. It can present at birth or late childhood and has variable presentations and severity. It consists of a heterogeneous group of disorders characterized by defective neuromuscular junction (NMJ) transmission. Muscle weakness is common in patients with CMS, but other clinical presentations depend on the genetic defect. CMS is associated with mutations of genes at NMJ, involving the acetylcholine receptors (AChR) subunits. Here, we present the case of a 14-month-old child who presented with refractory seizures and respiratory depression requiring prolonged ventilation and tracheostomy. His whole exome sequencing (WES) showed a variant in the homozygous state in the CHAT gene. Pathogenic variants in this gene are associated with autosomal recessive presynaptic congenital myasthenic syndrome type 6. He did not have any family history of myasthenia gravis and showed marked improvement after starting pyridostigmine despite being started late.

This case report has been accepted for poster presentation at the Oman Genetic Society for Genetic Medicine Scientific Research Day, which was held on the 5^th^ of December 2024.

## Introduction

Congenital myasthenia syndrome (CMS) is a congenital disorder that affects muscles and causes fatigability. It is an inherited disorder caused by different gene mutations where mutation in the CHRNE gene is the most common [1,2]. CMS affects neuromuscular transmission; most symptoms appear within several days after birth. However, it can present later in early childhood. Acetylcholine receptor (AChR) at the neuromuscular junction is impacted by genetic mutations linked to CMS linked to the CHRNE gene. CMS can specifically result from mutations in the AChR's epsilon subunit, which is encoded by the CHRNE gene [3]. CMS is characterized by variable presentation and severity, which range from minor symptoms to progressive disabling weakness. CMS is a rare disease and its diagnosis depends on clinical findings, measurement of ACHR antibodies, electromyography, and genetic testing. In this case report, we aim to emphasize the variable presentation of CMS as our case presented with refractory seizures and respiratory depression requiring frequent intubation and tracheostomy.

## Case presentation

The patient is a 14-month-old male child, who was born at 37 weeks of gestation by emergency cesarean section to a gravida 4, parity 3 mother who had pregnancy-induced hypertension and was on labetalol and had gestational diabetes mellitus that was managed on diet. He cried immediately after birth with an Apgar score of 6 and 8 at 1 and 5 minutes, respectively, and his birth weight was 2.7 kg. His systemic examination was normal with no dysmorphic features. Cord blood gas was normal. The parents are second-degree cousins and already had three normal children. There was no family history of convulsions or any other congenital diseases. A few minutes after birth, he became lethargic with a weak cry. He had good respiratory effort initially but started to have desaturations for which he was started on non-invasive ventilation. Later, he developed convulsions and became more lethargic so was intubated for airway protection. He had refractory seizures, which required multiple anticonvulsant medications to be controlled. He was kept on phenobarbitone 5 mg/kg/day divided into bis in die (BID) dose, levetiracetam 16 mg/kg/dose BID, and pyridoxine 40 mg once daily (OD). The convulsions were described as lip-smacking and cyclic movement with changes in heart rate and saturation. He also had excessive jitteriness. He was started on antibiotics for presumed sepsis, initially ampicillin and gentamycin but later upgraded to cefotaxime and cloxacillin for a total of seven days. He also received intravenous (IV) acyclovir for eight days for a presumed viral infection. Blood and cerebrospinal fluid (CSF) cultures were reported as normal. The CSF viral panel was negative. Initial metabolic workup was normal with normal blood gases, blood sugar, urine ketones, ammonia, and lactate. Tandem mass spectrometry test and creatinine kinase were also normal and deoxyribonucleic acid (DNA) was sent to the tertiary center to rule out any genetic disease. Head ultrasound (US) was normal and echocardiography showed normal heart structure and function. The child had mild stridor in the early days of admission, which spontaneously disappeared. He was subsequently weaned from the ventilator and could be extubated on day five of life.

He was observed to have hypertonia initially and continued to have episodes of lip-smacking and cyclic movements, which were brief at times but sometimes prolonged requiring loading doses of phenobarbitone. He was transferred to a higher center for magnetic resonance imaging (MRI), electroencephalography (EEG), and a multidisciplinary approach. At the tertiary care center, he was seen and evaluated by multiple teams. He was evaluated by the neurology team and EEG was initially normal, therefore levetiracetam was discontinued. Pyridoxine was also stopped and continued on phenobarbitone because he continued to have brief episodes of lip-smacking and cyclic movement. Whole exome sequencing (WES), which is a comprehensive genetic test that analyzes the protein-coding regions (exons) of all human genes, was sent to another country to rule out genetic disorders. He was also evaluated by the gastroenterology team where an impression of gastroesophageal reflux disease (GERD) was unlikely. He was referred to the speech therapist because he was on nasogastric tube (NGT) feeding and was not tolerating oral feeds. The assessment revealed mild hypersensitivity with absent sucking reflux. He attempted drops of milk on his lips and noted tasting and laryngeal elevation suggestive of aspiration. He was referred to an occupational therapist and examination showed intact oral motor structure with mild hypersensitivity on the palate, and hypotonic sucking with poor latching and tongue movement so sessions for oral motor stimulation were started. He was discharged on oral feeding and advised to keep 45 degrees while feeding. He was discharged at the age of one month on room air and NGT feeding.

The infant presented at the age of three months to the local hospital with a history of vomiting, choking, and gasping that required endotracheal intubation. He was escorted to a secondary-level hospital where he was admitted to the pediatric intensive care unit (ICU) and extubated second day of admission. He was found to have a failure to thrive with choking episodes so restarted on NGT feeding and continued on phenobarbitone. He was discharged on NGT feeding, and phenobarbitone in good condition.

The infant was admitted soon after discharge to the local hospital with the same complaint and was taken to a tertiary care hospital for re-evaluation of choking and recurrent seizures. He was again reevaluated by multidisciplinary teams; He was evaluated by the gastroenterology team who emphasized that the diagnosis of GERD is unlikely. However, his assessment revealed breathing-swallowing discoordination, which was causing microaspiration. Brain MRI and EEG were repeated and were normal. He was discharged home with follow-up appointments.

**Figure 1:**
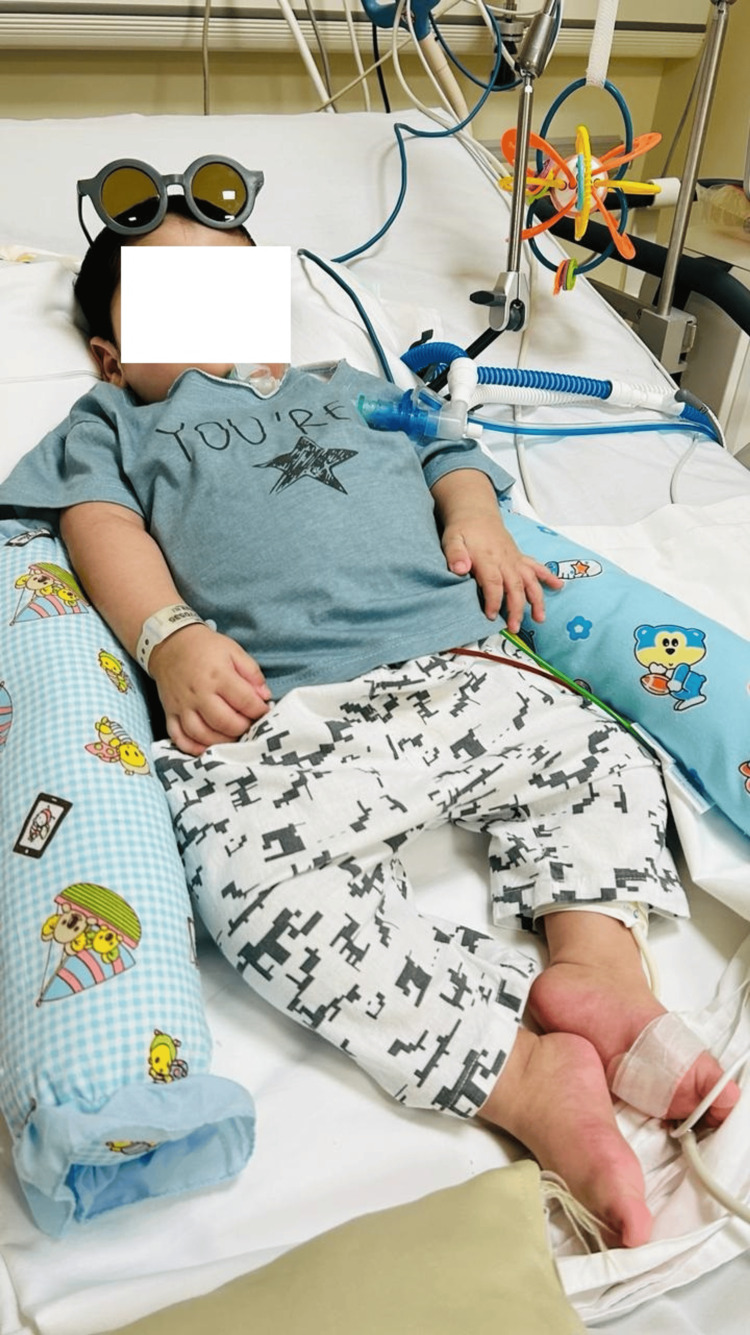
Picture of the child after tracheostomy

**Figure 2:**
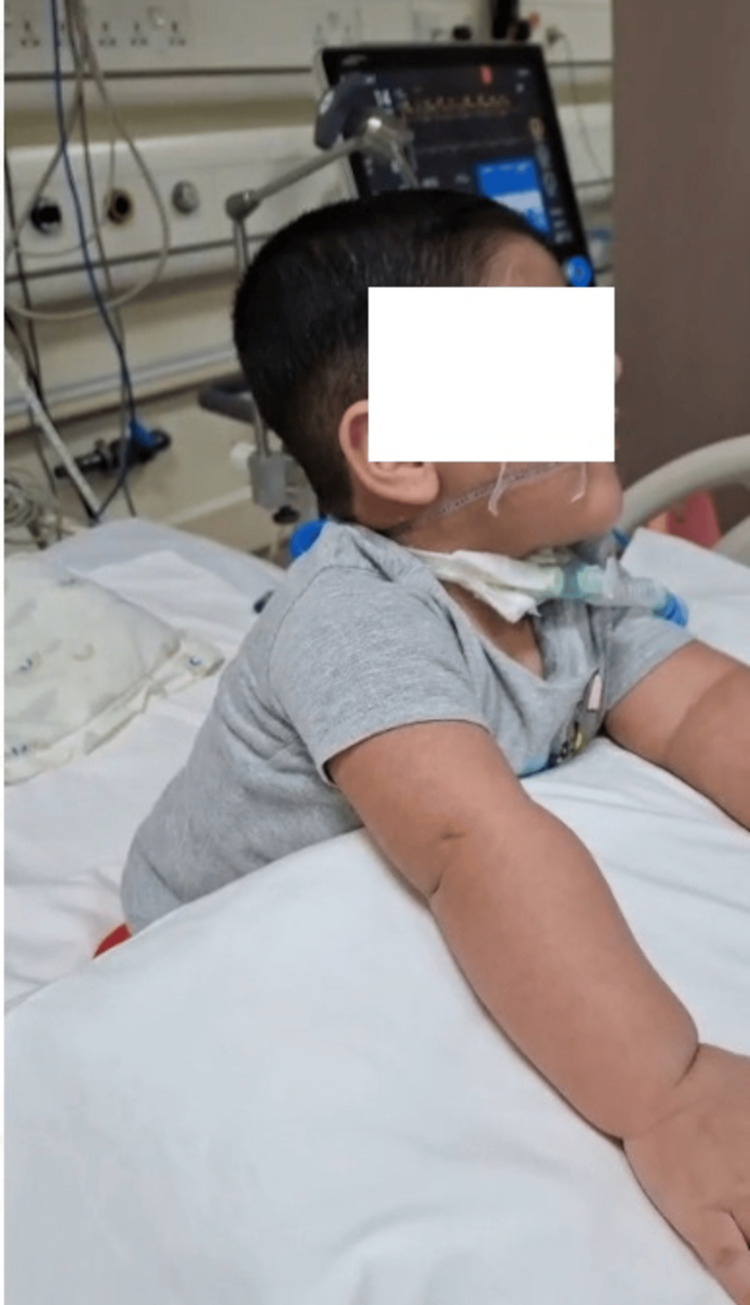
Picture of the child after definitive diagnosis and treatment, sitting with support, with reduced ventilatory settings

He continued to be admitted frequently with a chest infection and aspiration with apnea and seizures. He was vaccinated at the age of six months after which he developed a fever and respiratory distress. He was taken to a local hospital where he had severe respiratory distress and required intubation and was escorted to secondary hospital service where he was admitted to the ICU and tried extubation several times but each time failed because of desaturations, bradycardia, and apneas with convulsions. His anticonvulsant medications were adjusted. He was continued on levetiracetam 16 mg/kg/dose BID and carbamazepine was added at a dose of 7.5 mg/kg/dose BID. All basic investigations were normal and he was again referred to tertiary care for further investigations. He was seen by a pulmonologist who performed a bronchoscopy, which was normal. Other teams also evaluated and the impression was the same and to wait for the WES results. He was referred to the local hospital where he again tried extubation but failed. Finally, a tracheostomy was done due to his recurrent respiratory deterioration (Figure 1). The WES results were available at the age of 11 months, and it revealed an autosomal recessive presynaptic congenital myasthenia syndrome type 6. He was started on pyridostigmine 4 mg/kg/dose every sixth hour after which he showed marked improvement in his tone and level of consciousness. He started to gain some milestones like head control and social smile. His ventilator setting could be reduced but continued to be ventilator-dependent till the time of writing this report (Figure 2).

## Discussion

Myasthenia gravis is a chronic autoimmune neuromuscular disorder. It is characterized by easy fatigability of striated muscles like the eyelid, extraocular, and swallowing muscles. CMS can present at birth or early infancy with symptoms like ptosis, external ophthalmoplegia, difficulty in swallowing, hypotonia, weak cry, respiratory failure, facial weakness, and episodic apnea [4]. Acute life-threatening events have been reported as a presentation for CMS [5,6]. Recurrent apnea in infancy is a distinctive presentation of the presynaptic CMS, which is usually because of a mutation in ChAT [7]. CMS are almost always permanent disorders where there is no spontaneous improvement [8]. Most of these syndromes are transmitted as recessive traits but the slow channel syndrome is transmitted as an autosomal dominant trait. Most of these syndromes respond to cholinesterase inhibitors but in some forms, the symptoms and signs worsen. The genetic mutation is known in less than 50% of patients with CMS. Of all the patients with CMS, 85% have a mutation of DOK 7, and 5% of patients have a mutation of ChAT. Mutation of the COLQ gene has been identified in 10% of cases with CMS [9,10].

Typical clinical features are used to make the diagnosis. These include the following: a compound muscle action potential (CMAP) decrement on low-rate repetitive nerve stimulation; repetitive CMAP after single stimulation (double response to single nerve stimulation, which is typical of slow-channel CMS and endplate acetylcholinesterase deficiency/COLQ CMS); abnormal jitter on single-fiber electromyography (EMG); no clinical response to plasma exchange or other immunosuppressants; a positive response to acetylcholinesterase in most but not all cases; lack of serum AChR or muscle-specific kinase (MuSK) antibodies; the absence of typical stigmata of congenital myopathies on muscle biopsy; and genetic confirmation of a defect in one of the genes known to cause a CMS [4].

When a particular CMS has typical features, single gene testing is performed. However, a multigene panel or genomic testing is suggested when there are fewer typical clinical clues. Most CMS cases respond well to treatment with acetylcholinesterase inhibitors (pyridostigmine and 3,4-diaminopyridine), except fast-channel and DOK7 CMS. In CMS with DOK7 and COLQ defects, albuterol and ephedrine work wonders. Some patients with slow-channel CMS benefit from fluoxetine [11].

With correct diagnosis and compliance with medications and follow-ups, the long-term prognosis in children with CMS is fairly stable. Several measures that could potentially improve the quality of life in these patients are regular follow-ups, taking medications as indicated regularly, physical therapy to improve muscle strength and facilitate movements, and occupational therapy for performing daily activities.

Several challenges exist in diagnosing CMS in resource-limited settings. These include limited access to various diagnostic modalities, lack of training and expertise, the non-availability of a neurologist and genetic expert, and economic constraints. Improving healthcare infrastructure, raising awareness among clinicians, lowering the cost, and increasing the accessibility of diagnostic equipment are all necessary to meet these challenges. Genetic counseling in CMS is very essential. It helps in accurate diagnosis by differentiating CMS from other similar conditions, family planning, personalized treatment, and providing emotional support to the family members of the patients with CMS.

## Conclusions

This case report emphasizes early investigation, especially genetic testing and counseling, so that prompt treatment can be initiated to prevent recurrent hospitalization and unwanted complications. Diagnosis of CMS required meticulous clinical examination, a high sense of clinical suspicion, an early sample for advanced genetic testing like WES, and multidisciplinary care. Even though a possibility of complete improvement is unlikely, early diagnosis with genetic testing could facilitate the initiation of appropriate medications, thus leading to a better quality of life.
